# Impact of Type 2 Diabetes on Oncologic Outcomes of Hepatocellular Carcinomas in Non-Cirrhotic, Non-alcoholic Steatohepatitis: a Matched-Pair Analysis

**DOI:** 10.1007/s11605-020-04628-0

**Published:** 2020-05-06

**Authors:** Adrian T. Billeter, Philip C. Müller, Thomas Albrecht, Stephanie Roessler, Moritz Löffler, Anastasia Lemekhova, Arianeb Mehrabi, Beat P. Müller-Stich, Katrin Hoffmann

**Affiliations:** 1 Department of General-, Visceral- and Transplantation Surgery, University Hospital Heidelberg, Im Neuenheimer Feld 110, 69120 Heidelberg, Germany; 2 Institute of Pathology, University Hospital Heidelberg, Heidelberg, Germany

**Keywords:** NASH, NAFLD, HCC, Non-cirrhotic HCC, Diabetes, Type 2 diabetes mellitus

## Abstract

**Background:**

Non-alcoholic steatohepatitis (NASH) associated hepatocellular carcinomas (NASH-HCC) are increasing. NASH-HCC often develop**s** in the fibrotic liver. Several analyses report conflicting results regarding the outcome of non-cirrhotic NASH-HCC. Furthermore, type 2 diabetes (T2D) is considered a risk factor for poor survival. The aim of this study was to investigate oncological outcome**s** of non-cirrhotic NASH-HCC and the impact of T2D.

**Methods:**

Patients with non-cirrhotic NASH-HCC with T2D as determined by an expert pathologist conducting histological slide review were matched for risks factors for poor outcome (age, gender, body mass index) with patients with NASH-HCC without T2D. These patients were th**e**n matched 1:1 with HCC**s** of other underlying liver diseases with and without T2D. Oncological outcomes were assessed using Kaplan-Meier curves.

**Results:**

Out of 365 HCCs resected between 2001 and 2017, 34 patients with non-cirrhotic NASH-HCC were selected (17 with T2D, 17 without T2D) and matched with 26 patients with hepatitis-HCC and 28 patients with alcohol-related HCC. Oncological risk factors such as tumor size, resection margin, and vessel invasion were comparable. There was no difference in overall survival (5-year survival 71.3% for NASH-HCC, 60.4% for hepatitis-HCC, 79.9% for alcohol-HCC). NASH-HCC was associated with longer disease-specific survival than hepatitis-HCC (5-year 87.5% vs. 63.7%, *p* = 0.048), while recurrence-free survival was identical. T2D had no impact on oncological outcomes in either liver disease.

**Conclusion:**

Non-cirrhotic NASH-HCC ha**s** outcomes comparable with other underling etiologies. Despite a lack of cirrhosis, patients with non-cirrhotic NASH-HCC have the same risks of HCC recurrence as patients with cirrhotic liver disease of other etiologies.

## Introduction

Non-alcoholic fatty liver disease (NAFLD) and non-alcoholic steatohepatitis (NASH) are chronic liver diseases that may progress into cirrhosis or hepatocellular carcinoma (HCC).^1, 2^ Type 2 diabetes mellitus (T2D) and obesity are risk factors for NAFLD/NASH, as the three diseases are strongly associated. The main risk of NAFLD/NASH is the eventual development of other liver-related diseases, such as liver cirrhosis and hepatocellular carcinoma (HCC), as well as non-liver-related comorbidities, such as cardiovascular disease.^3, 4^ Several studies have shown that T2D is a strong risk factor for the progression of NAFLD and the development of HCC already in the fibrotic liver but not yet cirrhotic liver.^3, 5, 6^ Recent studies have also investigated the role of T2D in liver diseases with other underlying factors, such as hepatitis-related HCC or alcoholic liver disease, and have shown that T2D increases the risk of developing HCC, independent of the type of underlying liver disease.^5, 7, 8^ However, the impact of T2D on survival and recurrence remains a subject of debate. Furthermore, most studies until now have been based on large administrative databases with little or no matching for patient baseline characteristics. Also, while some studies indicate that NASH-associated HCC in particular often occurs during the fibrotic stage of liver disease without cirrhosis, few studies have exclusively investigated HCC in the non-cirrhotic liver.^9, 10^ Lastly, in previous studies, the histology of the adjacent liver tissue was not re-reviewed by an expert pathologist.

The aim of this study was to investigate the oncological outcome**s** of HCC in non-cirrhotic patients with NASH as confirmed by histological slide review by an expert liver pathologist. The oncological outcomes of these non-cirrhotic, NASH-associated HCCs were compared with matched patients with hepatitis or alcoholic liver disease related HCCs. The impact of T2D on oncologic outcome was investigated in patients matched for baseline characteristics. With this approach, we ensured that the outcome of non-cirrhotic NASH-HCC and the actual impact of T2D on HCC-development were assessed with the least possible bias.

## Patients and Methods

**Fig. 1:**
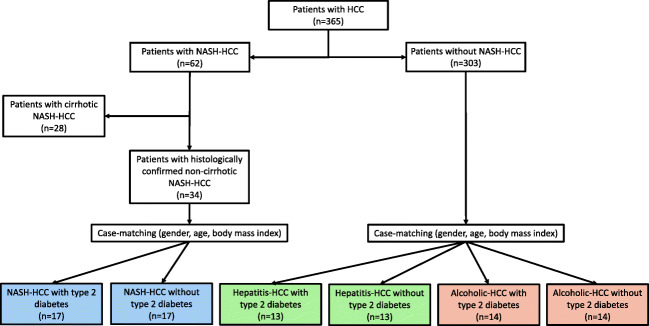
Flow chart of patient selection

The focus of this study is to compare closely matched patients with histologically confirmed non-cirrhotic NASH-HCC with HCCs of other etiologies, as well as to investigate the impact of T2D without confounding factors such as BMI, age, and gender. To achieve that goal, patients were strictly selected as outlined below. This study was approved by the ethics committee of our institution and conducted in accordance with the declaration of Helsinki and its latter amendments.^11^ Patients with HCC operated on between October 1, 2001, and December 31, 2017, were enrolled. Data were obtained from a prospectively maintained database. Patients with a diagnosis of NASH-related HCC were identified and further divided into groups of those with and without T2D (Fig. 1). Diagnosis of T2D was based on patient history, preexisting diagnosis from the referring primary care physician/hepatologist, and a separate evaluation by our internal medicine department. Furthermore, to confirm that patients did not have undocumented but medically treated preexisting T2D, pre- and perioperative medication was crosschecked to rule out any confounding factors or postoperative derangement of blood sugar requiring glucose lowering medication. Similarly, T2D diagnosis was confirmed by the postoperative use of blood sugar lowering medication. Patients with T2D and NASH-related HCC were then matched 1:1 with those with NASH-related HCC without T2D. Matching criteria were gender, age (± 3 years), and body mass index (BMI). Based on these patients with NASH-related HCC, patients with HCC related to other underlying liver disease (non-NASH) were also matched using the same criteria. The patients with non-NASH-related HCC were also divided into groups with and without T2D. Lastly, since this work focuses on oncological outcomes, all patients who died prior to discharge were excluded.

### Pathological Reassessment of Liver Histology

An expert pathologist re-assessed the liver histology of patients with NASH-related HCC. The presence of NASH was confirmed and SAF-score as described by Bedossa et al. was used to exclude other liver diseases such as an iron storage disorder, M. Wilson, or any other rare diseases.^12^

### Surgical Care

All patients underwent preoperative contrast-enhanced computer tomography (CT) and/or Primovist magnetic resonance imaging (MRI) to assess their liver lesions. In cases where imaging findings were unclear, a biopsy was performed to rule out any other basis for the lesions. All treatment decisions were made during regular meetings of the interdisciplinary tumor board at the Heidelberg Liver Cancer Center. Patients with liver cirrhosis (child B and C) were not considered candidates for surgical resection. The surgical strategy was determined by the responsible senior surgeon.

### Laboratory Parameters and Outcome Assessment

Preoperative liver function tests, including the aspartate transaminase (AST), alanine transaminase (ALT), alkaline phosphatase (AP), and γ-glutamyl transferase (γ-GT), liver syntheses parameters such as serum choline-esterase (CHE), serum total bilirubin, quick, and albumin, and renal retention parameters such as serum creatinine and serum urea were analyzed. Patients were then followed through our hepatobiliary outpatient clinic or the national center for tumor disease. Primary care providers performed follow-up for long-distance patients who could not come to the clinic. Recurrence-free survival was defined as the time interval to recurrence. Overall mortality, as well as HCC-related mortality (disease-specific survival), was also determined.

### Statistical Analysis

Continuous data are reported as median and interquartile range (IQR). Categorical data are reported as frequencies (*n*) and proportions (%). Continuous variables were compared with the Mann–Whitney U test. Differences among proportions derived from categorical data were compared using Fischer’s Exact or Pearson χ2 tests, as appropriate. Kaplan-Meier curves were used to estimate overall and recurrence-free survival. The level of significance was set at a *P* value of ≤  0.05. Statistical analysis was performed with IBM SPSS software (version 25.0; SPSS Inc., Armonk, NY).

## Results

### Selection of Patients

An overview of patient selection is provided in Fig. 1. Out of 365 patients with HCC, 62 were found to have NASH-related HCC. Of these, 17 had T2D (NASH+T2D) and no liver cirrhosis and were then matched with 17 patients with NASH-related HCC without T2D (NASH-T2D) and no liver cirrhosis. Thirteen patients with hepatitis-HCCs with T2D and 13 patients without T2D as well as 14 patients with alcoholic-HCCs with T2D and 14 without T2D were matched using the same criteria as for the NASH patients. This very stringent approach resulted in a highly selected group of patients with very similar characteristics (Table 1). Except for the Charlson comorbidity index (CCI), there were no significant differences among the patients regardless of the underlying liver disease, presence of T2D or oncological outcome parameters. The median follow-up time was 38.7 months (10.1–70.9).

**Table 1:** Baseline characteristics

	NASH-T2D (*n* = 17)		NASH+T2D (*n* = 17)		*P*	Hepatitis-T2D (*n* = 13)		Hepatitis+T2D (*n* = 13)		*P*	ALD-T2D (*n* = 14)		ALD+T2D (*n* = 14)		*P*	HCC-T2D (*n* = 44)		HCC+T2D (*n* = 44)		*P*
Demographics																				
Male sex	16	(94)	16	(94)	1.000	13	(100)	13	(100)	1.000	14	(100)	14	(100)	1.000	43	(98)	43	(98)	1.000
Age, years	68	(65–72)	69	(67–72)	0.708	67	(62–72)	68	(65–75)	0.243	71	(65–73)	68	(62–72)	0.265	68	(63–74)	68	(64–73)	0.748
BMI, kg/m^2^	28.0	(24.5–33.3)	26.5	(24.0–28.8)	0.551	25.2	(22.5–29.2)	26.3	(25.3–29.4)	0.410	27.3	(26.2–30.6)	26.3	(23.3–30.9)	0.260	28.0	(25.8–32.2)	26.1	(24.1–28.9)	0.549
Liver cirrhosis	0	(0)	0	(0)	1.000	9	(82)	6	(55)	0.361	10	(91)	8	(62)	0.166	19	(49)	14	(34)	0.186
CCI	5	(4–7)	7	(5–8)	0.024	5	(5–7)	7	(6–9)	0.010	7	(5–8)	7	(5–8)	0.164	6	(5–7)	7	(6–8)	0.038
Preoperative laboratory values																				
AST, U/l	33	(24–53)	27	(18–33)	0.193	61	(52–120)	47	(23–80)	0.039	40	(23–60)	36	(27–43)	0.427	45	(27–65)	28	(20–59)	0.027
ALT, U/l	35	(29–50)	33	(18–45)	0.182	65	(52–109)	30	(21–97)	0.004	39	(26–53)	27	(20–41)	0.210	44	(31–61)	33	(19–52)	0.003
Bilirubin, mg/dl	0.50	(0.38–0.98)	0.45	(0.33–0.58)	0.140	0.70	(0.43–0.95)	0.95	(0.55–1.32)	0.295	0.80	(0.58–1.13)	0.60	(0.50–1.00)	0.094	0.65	(0.48–1.00)	0.50	(0.40–0.95)	0.166
Quick, %	97	(91–107)	99	(92–101)	1.000	88	(77–98)	89	(82–97)	0.769	89	(80–99)	98	(78–100)	0.830	91	(86–101)	97	(85–101)	0.822
Platelets, G/l	261	(223–328)	213	(137–257)	0.099	126	(86–147)	180	(120–231)	0.320	187	(142–302)	208	(160–307)	0.603	203	(139–259)	198	(135–257)	0.792
AP, U/l	95	(82–176)	104	(85–113)	0.958	89	(73–140)	85	(68–151)	0.695	95	(71–153)	123	(107–223)	0.137	94	(76–118)	100	(74–122)	0.524
γ-GT, U/l	160	(60–282)	56	(38–128)	0.024	103	(36–214)	99	(47–238)	0.797	273	(52–512)	208	(53–319)	0.689	83	(43–293)	63	(39–144)	0.232
ChE, U/l	7.8	(5.6–9.7)	7.0	(5.8–8.0)	0.880	5.1	(4.2–7.1)	5.9	(4.3–8.6)	0.478	5.9	(4.1–7.9)	5.0	(2.7–7.3)	0.810	6.5	(5.0–8.2)	6.8	(5.1–8.0)	0.666
Albumin, g/l	43	(41–45)	42	(35–43)	0.191	41	(38–44)	41	(37–45)	0.630	41	(39–45)	40	(38–42)	0.910	43	(39–45)	42	(37–43)	0.594
Creatinine, mg/dl	0.83	(0.67–0.99)	1.01	(0.83–1.06)	0.181	0.77	(0.69–0.84)	0.83	(0.77–1.05)	0.091	1.04	(0.78–1.23)	0.79	(0.66–1.00)	0.094	0.89	(0.76–1.08)	0.92	(0.77–1.05)	0.239
Urea, mg/dl	31	(29–44)	36	(29–45)	0.375	33	(26–40)	40	(35–47)	0.091	28	(23–52)	33	(24–40)	1.000	33	(28–43)	38	(31–43)	0.188
Histology																				
Number of lesions	1	(1–1)	1	(1–1)	1.000	1	(1–2)	1	(1–2)	0.928	1	(1–1)	1	(1–1)	0.693	1	(1–1)	1	(1–1)	0.599
Tumor size, cm	6.0	(3.4–8.5)	3.5	(2.4–4.4)	0.170	3.2	(2.9–4.8)	2.2	(1.2–8.2)	0.392	3.5	(1.5–4.5)	4.7	(3.1–8.1)	0.094	3.5	(2.8–6.5)	3.1	(1.3–4.7)	0.752
Vascular invasion	8	(47)	4	(24)	0.203	2	(15)	3	(23)	0.263	2	(14)	4	(29)	0.591	12	(27)	11	(25)	0.405
Histologic grade					0.435					0.525					0.163					0.410
G1	1	(6)	3	(19)		0	(0)	1	(9)		1	(8)	1	(8)		2	(5)	5	(13)	
G2	11	(69)	11	(69)		10	(77)	9	(82)		10	(77)	5	(42)		31	(74)	25	(64)	
G3	4	(25)	2	(13)		2	(15)	1	(9)		2	(15)	6	(50)		8	(19)	9	(23)	
G4	0	(0)	0	(0)		1	(8)	0	(0)		0	(0)	0	(0)		1	(2)	0	(0)	
R-1 resections	0	(0)	3	(18)	0.227	1	(8)	1	(8)	1.000	1	(8)	1	(8)	1.000	2	(5)	5	(12)	0.433

Data are given as *n* (%) and median (IQR)

*ALT*, alanin-aminotransferase; *AST*, aspartat-aminotransferase; *BMI,* body mass index; *CCI*, Charlson comorbidity index; *ChE*, cholinesterase

### Histological Reassessment of Liver Tissue

All patients had NASH with inflammatory activity and ballooning. Furthermore, none of the patients had cirrhosis with a maximum fibrosis score of 3. The median inflammatory activity score was 2 (range 1–2) for patients with NASH+T2D and 1 (1–2) for patients with NASH–T2D (*p* = 0.192). The median fibrosis score was 2 (1–2) for patients with NASH+T2D and 1 (1–2) for patients with NASH–T2D (*p* = 0.264).

### NASH vs. Hepatitis and Alcoholic Liver Disease

Patients with NASH underwent more extensive liver resections and less segmental resections than patients with hepatitis (*p* < 0.001) or alcoholic liver disease (*p* = 0.003, table 2). Although there was no difference in major overall complications (whether surgical or non-surgical), when compared with the hepatitis and alcoholic liver disease group (*p* = 0.193 and *p* = 0.341, respectively), the surgical approach for NASH patients resulted in more surgical complications (and bile leaks in particular) than the hepatitis and alcohol liver disease group (*p* = 0.004 and *p* = 0.047, respectively). On the other hand, the NASH patients had a lower rate of non-surgical complications compared with both the hepatitis and alcohol liver disease groups (*p* = 0.003 and *p* = 0.001, respectively).

**Fig. 2:**
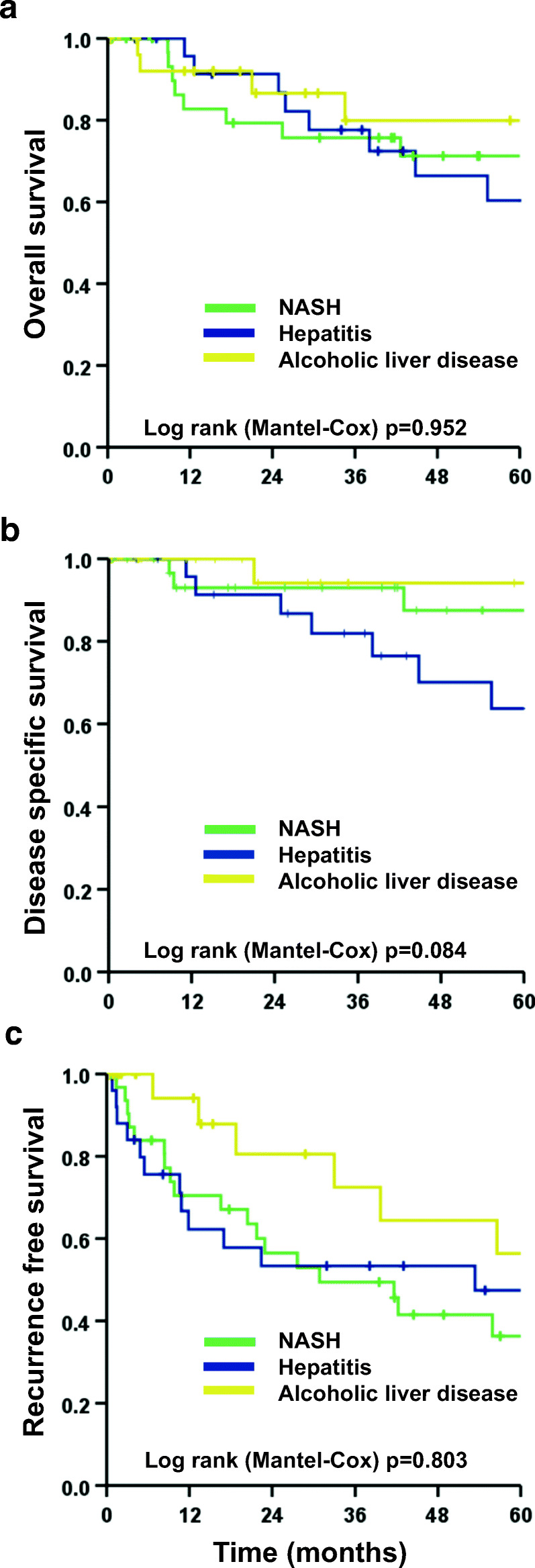
Kaplan-Meier plots of overall (**a**), disease-free (**b**), and recurrence-free survival (**c**) for NASH, hepatitis, and alcoholic liver disease

There was no difference in overall survival among NASH-HCC (1-, 3-, and 5-year survival of 82.8%, 75.7%, and 71.3%), hepatitis-HCC (1-, 3-, and 5-year survival of 95.7%, 77.6%, and 60.4%), and alcoholic-HCC (1-, 3-, and 5-year survival of 92.0%, 79.9%, and 79.9%, *p* = 0.952, Fig. 2a). Regarding disease-specific survival, NASH had a significantly better disease-specific survival rate (1-, 3-, and 5-year of 93%, 93%, and 87.5%) than hepatitis-HCCs (1-, 3-, and 5-year disease-specific survival rate of 95.7%, 81.9%, and 63.7%, *p* = 0.048) but was similar to alcoholic-HCCs (1-, 3-, and 5-year disease-specific survival rate of 100%, 94.1%, and 94.1%; *p* = 0.499, Fig. 2b). Recurrence-free survival was comparable between NASH-HCC (1-, 3-, and 5-year rates of 70.5%, 49.4%, and 36.3%), hepatitis-HCC (1-, 3-, and 5-year rates of 62.3%, 53.4%, and 47.4%, *p* = 0.974), and alcoholic-HCC (1-, 3-, and 5-year rates of 94.1%, 72.5%, and 56.4%, *p* = 0.486, Fig. 2c).

### NASH+T2D vs. NASH-T2D

**Fig. 3:**
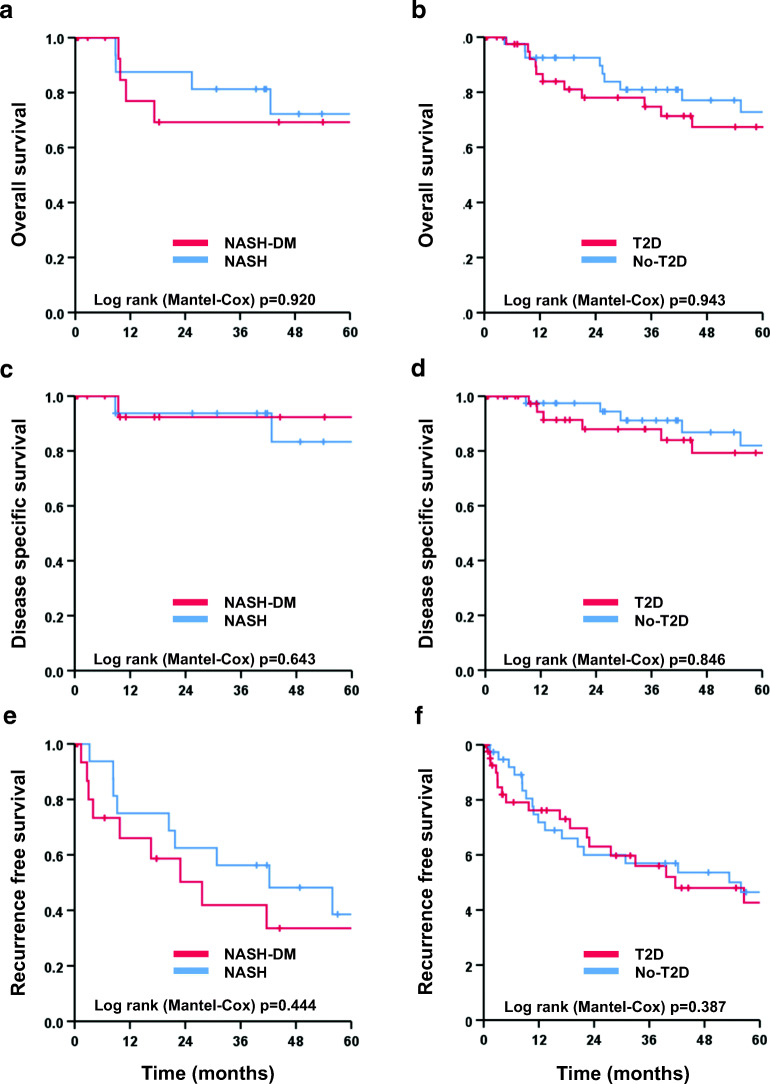
Kaplan-Meier plots of overall (**a**), disease-free (**c**), and recurrence-free survival (**e**) for patients with NASH and NASH-DM. Overall (**b**), disease-free (**d**), and recurrence-free survival (**f**) for patient**s** with and without type 2 diabetes mellitus are also depicted

Patients with or without T2D and NASH were well matched in terms of sex, age, and BMI (Table 1). No patients had cirrhosis. Preoperative liver function was comparable, except for a higher y-GT in the NASH-T2D group (160 U/l (60–282) vs. 56 U/l (38–128); *p* = 0.024). No difference was found in tumor grading, vascular invasion, or tumor size (Table 1). Nor was there any difference in frequency, severity (major/minor), or type (surgical/non-surgical) of postoperative complication (Table 2). Oncologic outcome was similar between the NASH-T2D and NASH+T2D group**s**, with 1-, 3-, and 5-year overall survival rates of 87.5%, 81.3%, and 72.2% for the NASH-T2D group and of 76.9%, 69.2%, and 69.2% for the NASH+T2D group (*p* = 0.920, Fig. 3a). Disease-specific survival was also comparable, at 93.8%, 93.8%, and 83.3% at 1-, 3-, and 5-years in the NASH-T2D group versus 92.3%, 92.3%, and 92.3% in the NASH+T2D group (*p* = 0.643, Fig. 3b). The 1-, 3-, and 5-year recurrence-free survival rates were 75.0%, 56.3%, and 38.6% for the NASH-T2D group and 66.0%, 41.9%, and 33.5% for the NASH+T2D group (*p* = 0.444, Fig. 3c).

**Table 2:** Perioperative characteristics

	NASH (*n* = 17)		NASH-T2D (*n* = 17)		*P*	Hepatitis (*n* = 13)		Hepatitis-T2D (*n* = 13)		*P*	ALD (*n* = 14)		ALD-T2D (*n* = 14)		*P*	No-T2D (*n* = 44)		T2D (*n* = 44)		*P*
Surgical procedures																				
Segment resection	5	(29)	10	(59)	0.166	13	(100)	11	(85)	0.480	12	(86)	11	(79)	1.000	30	(68)	32	(73)	0.816
Left hemihepatecomy	4	(24)	2	(12)	0.656	0	(0)	0	(0)	1.000	1	(7)	0	(0)	1.000	5	(11)	2	(5)	0.434
Right hemihepatecomy	7	(41)	3	(18)	0.259	0	(0)	1	(8)	1.000	1	(7)	3	(21)	0.596	8	(18)	7	(16)	1.000
Extended left hemihepatecomy	0	(0)	1	(6)	1.000	0	(0)	0	(0)	1.000	0	(0)	0	(0)	1.000	0	(0)	1	(2)	1.000
Extended right hemihepatecomy	1	(6)	1	(6)	1.000	0	(0)	1	(8)	1.000	0	(0)	0	(0)	1.000	1	(2)	2	(5)	1.000
Postoperative complications																				
Clavien-Classification^**$**^					0.480					0.809					0.270					0.272
Minor (I-IIIa)	3	(18)	6	(35)		5	(39)	6	(46)		6	(43)	6	(43)		14	(32)	18	(41)	
Major (IIIb-V)	6	(35)	4	(24)		2	(15)	1	(8)		4	(29)	1	(7)		12	(27)	6	(14)	
Surgical complications	5	(29)	4	(24)	1.000	0	(0)	0	(0)	1.000	2	(14)	0	(0)	0.481	7	(16)	4	(9)	0.521
Biliary leak	5	(29)	2	(12)	0.398	0	(0)	0	(0)	1.000	1	(7)	1	(7)	1.000	6	(14)	3	(7)	0.484
Postoperative hemorrhage	1	(6)	0	(0)	1.000	0	(0)	0	(0)	1.000	1	(7)	0	(0)	1.000	2	(5)	0	(0)	0.494
Liver failure	0	(0)	0	(0)	1.000	0	(0)	0	(0)	1.000	0	(0)	0	(0)	1.000	0	(0)	0	(0)	1.000
Re-operation	1	(6)	1	(6)	1.000	0	(0)	0	(0)	1.000	2	(14)	1	(7)	1.000	3	(7)	2	(45)	1.000
Non-surgical complications	4	(24)	2	(12)	0.656	7	(54)	7	(54)	1.000	9	(64)	7	(50)	0.704	20	(46)	16	(36)	0.516
Hospital stay, days	14	(9–26)	12	(8–15)	0.454	9	(8–19)	10	(7–16)	0.762	10	(8–18)	9	(8–13)	0.667	11	(8–20)	9	(7–13)	0.370
Recurrences																				
Local recurrence	8	(47)	8	(47)	1.000	8	(62)	5	(39)	0.217	4	(29)	5	(36)	1.000	20	(45)	18	(41)	0.851
Distant recurrence	3	(18)	2	(12)	1.000	1	(8)	4	(31)	0.161	1	(7)	2	(14)	1.000	5	(11)	8	(18)	0.738

Data are given as *n* (%) and median (IQR)

*ALD*, alcoholic liver disease; *T2D*, type 2 diabetes mellitus

^$^Refers to Dindo et al.

### T2D vs. No-T2D

Patients with and without T2D, independent of underlying liver disease type, were well matched for sex, age, and BMI (Table 1). Preoperative liver function tests showed slightly higher transaminases in patients without T2D (AST 45 U/l (27–65) vs. 28 U/l (20–59); *p* = 0.027 and ALT 44 U/l (31–61) vs. 33 U/l (19–52); *p* = 0.003) while all other liver and renal retention parameters were comparable. There was no difference in tumor size, histological grad**e**, or vascular invasion (Table 1). The occurrence, severity, and type of postoperative complications did not differ between patients with and without T2D (Table 2). Regarding oncologic outcomes, there were no differences in 1-, 3-, and 5-year overall survival rates (No-T2D: 92.5%, 80.9%, and 72.8% vs. T2D: 86.6%, 74.8%, and 67.4%; *p* = 0.943; Fig. 3d), disease-specific survival rate (No-T2D: 97.4%, 91.1%, and 82.0% vs. T2D: 94.3%%, 87.9%, and 79.3%; *p* = 0.846, Fig. 3e), and 1-, 3-, and 5-year recurrence-free survival rates (No-T2D: 71.9%, 57.0%, and 46.5% vs. T2D: 76.2%, 56.0%, and 42.7%; *p* = 0.387, Fig. 3f). Lastly, the presence of T2D did not impact recurrence, whether local or distant (Table 2).

### T2D in Hepatitis and Alcoholic Liver Disease

The baseline characteristics of patients with hepatitis-HCC and alcoholic-HCC with and without T2D did not differ in terms of tumor size, grade, and vascular invasion or in overall postoperative morbidity (Table 1). The 1-, 3-, and 5-year overall survival rates (Hepatitis-T2D: 100%, 72.7%, and 60.6% vs. Hepatitis+T2D: 90.9%, 81.8%, and 60.6%; *p* = 0.950 and ALD-T2D: 91.7%, 91.7%, and 91.7% vs. ALD+T2D: 92.3%, 71.2%, and 71.2%; *p* = 0.978), 1-, 3-, and 5-year disease-specific survival rates (Hepatitis-T2D: 100%, 90.9%, and 67.3% vs. Hepatitis+T2D: 90.9%, 81.8%, and 60.6%; *p* = 0.669 and ALD-T2D: 100%, 100%, and 100% vs. ALD+T2D: 100%, 90.0%, and 90.0%; *p* = 0.403), and the 1-, 3-, and 5-year recurrence-free survival rates (Hepatitis-T2D: 55.6%, 46.3%, and 37.0% vs. Hepatitis+T2D: 68.4%, 59.8%, and 59.8%; *p* = 0.635 and ALD-T2D: 87.5%, 75.0%, and 75.0% vs. ALD+T2D: 100%, 68.6%, and 34.3%; *p* = 0.106) were comparable, regardless of whether T2D was also present.

## Discussion

This study shows that patients with NASH-related HCC and non-cirrhotic livers have oncologic outcomes similar to those with HCCs due to other underlying liver diseases. The only detectable difference is a significantly better rate of disease-free survival compared with patients with hepatitis-HCCs. However, overall survival and recurrence-free survival is similar among all of the groups. Furthermore, T2D has no effect on the oncological outcome of HCC. Conversely, despite a lack of cirrhosis, patients with non-cirrhotic NASH-HCC have the same risks of HCC recurrence as patients with other underlying liver disease with cirrhosis. This finding highlights the cancerogenic environment in active NASH.

These findings shed light on the current literature, which maintains no consistent position on the role of T2D in HCC. This study is unique in that it investigates a clinical cohort via long-term follow-up and is further strengthened by the expert reassessment of histological data. Until now, most studies have used data from large registries, which innately pose a higher risk for bias. These database-driven studies describe an increase in NASH-related HCC and the need for liver transplantation due to NASH-cirrhosis, as well as anticipating that numbers will increase further,^13, 14^ while NAFLD is said to be associated with the highest rate of non-cirrhotic HCC.^15, 16^ In contrast, we did not observe any differences in overall survival and recurrence-free survival among the different underlying diseases and instead found that NASH-HCC was associated with better disease-free survival than hepatitis-HCC, although it was similar to alcoholic-HCC. Younossi et al. found that patients with NAFLD-HCC had worse overall survival and a lower likelihood of receiving a liver transplantation compared with HCC of other underlying causes. ^17^ Using the Surveillance, Epidemiology and End Results (SEER) database, patients were compared without adjustment for baseline differences, resulting in significant differences in age and comorbidities while no oncologic parameters such as liver histology, tumor size, or vascular invasion were available. This analysis was adjusted for baseline differences such as age and gender, but also for BMI and T2D, which are well-described risk factors for a worse prognosis. Importantly, the oncologic parameters were comparable. Hence the previously observed differences in survival might be related to differences in baseline characteristics. Another study using SEER data found that patients with NAFLD-HCC were older, more often female, had larger tumors, and had more metastatic disease than other etiologies, thus resulting in a lower rate of curatively intended for this group, in addition to a lower rate of survival after liver transplantation. However, in patients with resectable disease, survival was comparable with that of other liver diseases, with the exception of hepatitis B, which had a significantly higher survival rate.^18^ Similarly, Pais et al. also found that overall and recurrence-free survival did not differ due to underlying liver pathology.^16^

Regarding the higher surgical complication rate in the NASH cohort compared to patients with other HCC etiologies, we believe that this difference is primarily due to the higher rate of extended liver resections due to the lack of liver cirrhosis. It is well known that major liver resections pose a higher complication rate than atypical resections.^19^

The other aim of this study was to investigate the role of T2D on outcomes after HCC resection, as the available literature on this topic is scarce. Several large registry studies have found that T2D and metabolic syndrome are strong risk factors for the progression of liver disease into cirrhosis as well as the development of HCC.^3, 20, 21^ After resection, T2D seems to have no impact on disease-free survival.^22, 23^ However, Wang et al. observed an increased overall mortality in patients with T2D that was not due to HCC.^22^ Importantly, none of these studies specifically investigated NASH-HCC or outcomes specific to underlying liver pathology. Furthermore, as both of these studies investigating the impact of T2D on mortality involved Asian cohorts, data on the impact of T2D on HCC-related outcomes in Western populations remain scarce. We did not observe any effect of T2D on perioperative complication rates or long-term oncological outcomes in the NASH-HCC group or that of any other HCC-etiology. Furthermore, there were also no differences in surrogate oncological markers such as tumor size or vascular invasion in NASH-HCC±T2D without cirrhosis. Due to the design of this study, with its matched baseline characteristics such as age, gender, BMI, and other comorbidities that may influence outcomes, we can reliably determine that T2D seems not to have an effect on outcomes in resectable HCC, independent of underlying liver disease type.

Despite this finding, it is important to remember that all patients with NASH-HCC in this cohort had a non-cirrhotic liver. Schiffman et al. also found that patients with fibrotic livers have a similar recurrence rate as similar patients with cirrhosis.^24^ Mohamad et al. and Bengtsson et al. also found that overall mortality and recurrence were comparable in a Western cohort when non-cirrhotic NASH and other liver diseases were adjusted for age and treatment.^9, 25^ In contrast, three studies in Asian cohorts found that patients with non-cirrhotic NASH-HCC seem to have a more favorable outcome than those with other underlying liver diseases.^10, 18, 26^ Therefore, we propose that the risk of HCC recurrence in non-cirrhotic NASH livers, at least in Western patients, is similar to that of cirrhotic patients, and that close surveillance after curative resection should be maintained.

Another point that should be considered regarding NASH patients is the use of bariatric surgery to reduce the risk of HCC. Several analyses have shown that patients have a lower risk of developing HCC after bariatric surgery than those who have not had bariatric surgery.^27^ Furthermore, several studies have convincingly shown that bariatric surgery is able to improve existing NASH and restore liver histology back to normal in a large proportion of patients.^28^ However, no study thus far has investigated the effect of bariatric surgery on recurrence risk after HCC resection.

This study has several limitations, primarily the small cohort size and the long duration of patient recruitment. However, we purposefully chose to study a very select group of patients with non-cirrhotic NASH-HCC that were closely matched for several well-known risk factors (age, gender, and BMI) with non-NASH-HCC and similar oncologic risk factors. Furthermore, the groups were also matched from within, for concomitant T2D. Such a detailed analysis naturally reduces the number of patients available for study, but it also allows for studying the impact of very specific parameters, namely underlying liver pathology and T2D. The differences in NASH-HCC outcomes described in other studies are likely based on baseline differences in age, gender, and BMI and not due to the underlying liver disease. Regarding the duration of patient enrollment, the primary therapy options for resectable HCC did not change over this time, and adjuvant therapy options did not change much, either. Changes in perioperative assessment, postoperative care (with the introduction of fast track surgery principles), and better surveillance methods, however, may play a role. Nevertheless, the primary treatment option for resectable HCCs like those considered here has always been surgery, while the role of chemotherapy, especially as adjuvant therapy, remains strongly limited even today and does not apply to the patients investigated here. Lastly, we do not have data on the quality of preoperative or postoperative glycemic control. While we tried to address the lack of glucose or HbA1c measurements by cross-checking the need for pre- and postoperative glucose lowering medication, the impact of the quality of glycemic control on diabetes-related complications is unclear and data for micro- and macrovascular complications show no association between tight glycemic control and the development of diabetes-related complications.^29, 30^

## Conclusion

Resectable NASH-HCC**s** in non-cirrhotic livers in a Western cohort have outcomes comparable with those of resectable HCCs of other underling etiologies. However, despite a lack of cirrhosis, patients with non-cirrhotic NASH-HCCs have the same risks of HCC recurrence as patients with other underlying liver diseases with cirrhosis, thus highlighting the cancerogenic environment of active NASH. Overall survival is similar among NASH-HCC, hepatitis HCC, and alcoholic-HCC, although disease-specific survival is worse for hepatitis-HCC than for NASH-HCC and alcoholic-HCC. In the studied cohort, T2D does not influence perioperative or long-term oncological outcomes independent of type of underlying liver disease. However, these findings need to be confirmed with larger cohorts and similarly stringent matching criteria.
